# The Non-Specific Binding of Fluorescent-Labeled MiRNAs on Cell Surface by Hydrophobic Interaction

**DOI:** 10.1371/journal.pone.0149751

**Published:** 2016-03-01

**Authors:** Ting Lu, Zongwei Lin, Jianwei Ren, Peng Yao, Xiaowei Wang, Zhe Wang, Qunye Zhang

**Affiliations:** 1 Division of Endocrinology and Metabolism, Shandong Provincial Hospital affiliated to Shandong University, Jinan, China; 2 The Key Laboratory of Cardiovascular Remodeling and Function Research, Chinese Ministry of Education and Chinese Ministry of Health; The State and Shandong Province Joint Key Laboratory of Translational Cardiovascular Medicine, Qilu Hospital of Shandong University, Jinan, China; 3 Health Division of Guard Bureau, General Staff Department of Chinese PLA, Beijing, China; 4 Traditional Chinese Medicine Department, Jinan Firefighting Hospital, Jinan, China; University of Torino, ITALY

## Abstract

**Background:**

MicroRNAs are small noncoding RNAs about 22 nt long that play key roles in almost all biological processes and diseases. The fluorescent labeling and lipofection are two common methods for changing the levels and locating the position of cellular miRNAs. Despite many studies about the mechanism of DNA/RNA lipofection, little is known about the characteristics, mechanisms and specificity of lipofection of fluorescent-labeled miRNAs.

**Methods and Results:**

Therefore, miRNAs labeled with different fluorescent dyes were transfected into adherent and suspension cells using lipofection reagent. Then, the non-specific binding and its mechanism were investigated by flow cytometer and laser confocal microscopy. The results showed that miRNAs labeled with Cy5 (cyanine fluorescent dye) could firmly bind to the surface of adherent cells (Hela) and suspended cells (K562) even without lipofection reagent. The binding of miRNAs labeled with FAM (carboxyl fluorescein) to K562 cells was obvious, but it was not significant in Hela cells. After lipofectamine reagent was added, most of the fluorescently labeled miRNAs binding to the surface of Hela cells were transfected into intra-cell because of the high transfection efficiency, however, most of them were still binding to the surface of K562 cells. Moreover, the high-salt buffer which could destroy the electrostatic interactions did not affect the above-mentioned non-specific binding, but the organic solvent which could destroy the hydrophobic interactions eliminated it.

**Conclusions:**

These results implied that the fluorescent-labeled miRNAs could non-specifically bind to the cell surface by hydrophobic interaction. It would lead to significant errors in the estimation of transfection efficiency only according to the cellular fluorescence intensity. Therefore, other methods to evaluate the transfection efficiency and more appropriate fluorescent dyes should be used according to the cell types for the accuracy of results.

## Introduction

MicroRNAs (miRNAs) are small noncoding RNA gene products about 22 nt long and regulate the expression of target genes by complementarily binding to their 3 'untranslated region (3'UTR) [1]. MicroRNAs play important roles in almost all biological processes and the pathogenesis of various diseases including cancer, cardiovascular and endocrine diseases. For example, the expression of miR-125b, miR-145, miR-21 and miR-155 was abnormal and it was closely related to the progression, metastasis and prognosis of breast cancer [2]. Hyperglycemia promoted the development of diabetic complications by decreasing the expression of miR-1 in endothelial cells [3]. Another study found that the dysregulation of miRNAs expression in peripheral regulatory T (Treg) cells of newly diagnosed patients with Graves ‘disease was associated with the inhibition of retinoic acid signaling pathway and was the major cause of dysfunction of Treg cells [4]. Therefore, methods and technologies of miRNA research were studied in depth in order to better reveal the physiological and pathological significance of miRNAs. Currently, commonly used technologies in miRNA research included the detection of miRNA expression using gene chip, high throughput sequencing and quantitative PCR, discovery and verification of miRNA target genes using a dual luciferase reporter vector, high throughput sequencing of crosslinking immunoprecipitation (HITS-CLIP), et.al, wherein the transfection and tracing of miRNA mimic or inhibitor were the key technologies for studying miRNA function [5].

Cationic liposomes are the most commonly used method for nucleic acid transfection and can efficiently deliver nucleic acids into cells. Its advantages are convenient, economical, efficient and so on [6, 7]. The positively charged cationic liposomes and negatively charged nucleic acid sequences can form liposome-nucleic acid complexes with positive net charge by electrostatic interactions. Then these complexes bind to cell surfaces and enter the cells by endocytosis [8]. In some cells, such as primary cells and suspension cells, the transfection efficiency of cationic liposome-DNA complexes is very low. There are many reasons for the low transfection efficiency. For instance, the proliferation rate of primary cells is relatively low; the membrane composition of cells is different; the heparan sulfate proteoglycan on cell surfaces can interfere the binding of cationic liposomes-nucleic acid complexes to the cell membrane [9–11]. In addition, the fluorescent label is an important tracing technology and is widely used in real time PCR, flow cytometry, intracellular localization and detection of molecular interaction [12, 13]. Many fluorescent dyes can be used as markers. The cyanine fluorescent dye (Cy5 and Cy3) is composed of two indole rings and five or three methine groups. It can bind to nucleic acids and proteins in the ground state and its application is very wide. Its advantages include sharp absorption band, high extinction coefficient, less autofluorescence, great photostability and low pH sensitivity [14]. Carboxyfluorescein (FAM) is another commonly used fluorescein derivative and its advantages include convenient synthesis, stability in water and so on. FAM can bind to biological macromolecules by two carboxyl groups and be widely used for labeling proteins and nucleic acids in biomedical research [15, 16].

The transfection of fluorescently labeled RNA is one of the commonly used methods to study gene function. There were a lot of studies for optimizing the experimental parameters and were also some studies on its characteristics and mechanism. However, the mechanism of how fluorescently labeled RNA molecules enter cells has remained controversial. Some studies showed that the nucleic acid molecules labeled with cyanine fluorescent dye Cy3 could freely enter cells, but other studies suggested they could not enter cells without liposome and endocytosis [17]. The studies focusing on the specificity of transfection are still few so far. However, in some other studies, it was found that strong fluorescence signals were detected on cells when Cy5 or Cy3-labeled siRNA was electrically transfected into cells while the actual transfection efficiency was very low. There were similar results that Cy3-labeled RNA was transfected into Hela cells by lipofectamine [18, 19]. Despite this, the study of these phenomena and their mechanisms was still lacking. A studies suggested that there were nonspecific fluorescence signals when fluorescently labeled miRNAs were transfected into cells and the reason was that fluorescently labeled miRNAs were cleaved by RNAse in culture medium and the cleaved fragments with fluorescent labels diffused into the cells [20]. However, many studies have shown that the plasma miRNAs were tolerant to RNAse and were stable for long time [21, 22]. Obviously, more in-depth study was needed in order to clarify the characteristics and specificity of liposomal transfection with fluorescently labeled RNAs, especially miRNAs.

In this study, the miRNAs labeled with different fluorescence dyes were transfected into adherent and suspension cells by liposomal transfection reagent. Then, the characteristics, specificity and potential mechanisms of liposomal transfection of fluorescent-labeled miRNAs were studied. Our results showed that the miRNAs labeled with some fluorescent dyes could bind to the cell surface even without the formation of positively charged liposome-miRNA complexes. The hydrophobic interactions played important roles in this binding process. Moreover, this non-specific binding was associated with cell types, the structure and hydrophobicity of fluorescent dyes. These results implied that it would lead to significant errors in the estimation of transfection efficiency only according to the cellular fluorescence intensity when fluorescently labeled miRNAs were transfected into cells. Therefore, other methods to evaluate the transfection efficiency and more appropriate fluorescent dyes should be used for the accuracy of results.

## Materials and Methods

### Cell treatment and transfection

K562 and Hela cells were obtained from the American Type Culture Collection and were cultured in 1640 medium (Gibco, Life technology, USA) supplemented with 10% fetal bovine serum (Hyclone, Thermo Scientific, USA) in a humidified incubator in 5% CO_2_ at 37°C. For transfection of miRNA, 2.5x10^5^ K562 cells or 1x10^5^ Hela cells were seeded on each well of 12-well plate. After culturing for 24 hours or adherent Hela cells reached approximately 70% confluency, cells were transfected under optimized transfection conditions. Briefly, 3μl lipofection reagent (Lipofectamine RNAiMAX, Invitrogen, USA) was diluted in 50ul serum free medium Opti-MEM (Life Technologies, Carlsbad, CA, USA). Fluorescently labeled miRNAs (Cy5-miR363, Cy5-miR195, Cy5-miR1, FAM-miR195), short random sequence (Cy5-RS: 5’-UCACAACCUCC UAGAAAGAGUAGA-3’; FAM-RS: 5’-UUCUCCGAACGUGUCACGUTT-3’) (Ribobio, Guangzhou, China) were also separately diluted in Opti-MEM at working concentration (100nM). Part of them was mixed with diluted RNAiMAX reagent and was allowed to stand for 5min at room temperature to form positively charged liposome-miRNA complexes. A part of K562 or Hela cells were separately mixed with different liposome-miRNA complexes and cultured for different times. A part of K562 or Hela cells were treated only with fluorescently labeled miRNAs for different times. In some experiments, K562 and Hela cells were treated with RNaseA (1mg/ml) for 30min (or methanol/high salt buffer for 10min) after incubation with fluorescently labeled miRNAs with or without transfection reagent.

### Flow Cytometry analysis

Twenty four hours after different treatments, Hela cells were treated by trypsin (Gibco, Life technology, USA) and then Hela and K562 cells with different treatments were collected by centrifugation at 500 rpm for 5 minutes. After washing 3 times with phosphate buffer saline (PBS), the fluorescence signals of Cy5 and FAM were respectively detected at 633nm and 488nm wavelength by flow cytometry (BD FACSCalibur, USA). The data were analyzed by Flowjo Software 7.2 (Tree star, Inc. San Carlos, CA).

### Laser confocal microscopy detection

Twenty four hours after different treatments, the treated Hela and K562 cells were washed 3 times with PBS and then fixed with 4% paraformaldehyde for 30 min. After washing with PBS, some cells were directly analyzed by laser confocal microscope (LSM710, Carl Zeiss, Germany). The other cells were stained with 1ug/ml DAPI (4', 6-diamidino-2-phenylindole dihydrochloride) in methanol for 25 minutes at 37°C and then analyzed by LSM710. The fluorescence images of Cy5, FAM and DAPI were taken, respectively at 633 nm, 488 nm and 358 nm wavelengths. The total fluorescence intensity per cell (TFIPC) was calculated by ZEN 2009 Light Edition and Imagepro plus software (Media Cybernetics, USA).

### High salt wash

To destroy the electrostatic interactions, the treated Hela and K562 cells were respectively washed with 20mM Tris-HCL/2M NaCl pH7.6 buffer or 10mM KH_2_PO_4_ 350mM KCl pH5.0 buffer. After centrifugation and washing with PBS, the fluorescence signals of Cy5, FAM and DAPI in cells were analyzed using laser confocal microscopy or flow cytometry as described above.

### Real-time PCR

Total RNA of Hela and K562 cells with different treatments were isolated according to the protocol of TRizol reagent (Invitrogen, Carlsbad, CA). RNA concentration was measured by NanoDrop 2000 (Thermo Scientific, Hudson, NH). A part of total RNA was reversely transcripted using a miRCURY LNA Universal cDNA synthesis kit (Exiqon, Vedbaek, Denmark) following the manufacturer’s instructions. Quantitative real time PCR of miRNA was performed using Exiqon’s SYBR Green Master Mix, specific LNA PCR primer sets were purchased from Exiqon, human U6 was used as reference. A part of total RNA was reversely transcripted by a RevertAid First Strand cDNA synthesis kit (Thermo Scientific, Hudson, NH). Then, quantitative real time PCR was carried out using Maxima SYBR Green/ROX qPCR Master Mix (Thermo Scientific, Hudson, NH) to detect the expression of TWF1, a target gene of miR1. The primer sequence was as follows: sense: 5’ -GGTGTGGACACTAAGCATCAAACACTACAAGG-3’; anti-sense: 5’-ATCTATTTCCA ACTGCA CATAGTTGAGCTGTC-3’. Human β-actin was used as reference. MiRNA and mRNA was amplified and detected in the IQ5 iCycler (BioRad, Hercules, CA). Results were analyzed by the BioRad standard software (BioRad, Hercules, CA). Experiments were repeated independently for three times.

### MiRNA luciferase reporter assay

The sequences completely complementary to miR363 and miR195 were synthesized and inserted into the multiple cloning site of pGL3 vector (Promega, Madison, WI) to construct miRNA luciferase reporter vectors in accordance with the manufacturer's instructions. Then, these vectors were separately co-transfected with pRL-TK vector, which contains Renilla reniformis luciferase reporter gene and was used as an internal reference, into K562 and Hela cells using Nucleofector (Amaxa Biosystems, Cologne, Germany) as described in the manufacturer's protocol. Then, the transfected cells were incubated with Cy5-miR363 or FAM-miR195 with or without RNAiMAX reagent. The untreated cells were used as negative control and the cells only co-transfected with miRNA luciferase reporter vector and pRL-TK vector were used as positive control. Luciferase activity was assayed by Dual-Luciferase Reporter Assay System (Promega). The results were presented as 1-RRR (Relative Response Ratio). RRR = (firefly/Renilla of experimental sample–firefly/Renilla of negative control)/ (firefly/Renilla of positive control–firefly/Renilla of negative control). 1-RRR was positively correlated to the amount of intracellular miRNA. The smaller 1-RRR is, the less intracellular miRNA amount is, and vice versa. Experiments were repeated independently for three times.

### Surface hydrophobicity detection of cell membrane

The hydrophobicity of membrane surface of K562 and Hela cells was assayed using microbial adhesion to hydrocarbon (MATH) that was modified according to the protocol described previously [23]. Briefly, the cells were centrifuged and resuspended in PBS. The MATH assay hydrocarbon was a mixture of 50% v/v n-hexadecane/toluene (Sigma Chemical Louis, MO). In a clean borosilicate glass tube, 300 μL hydrocarbon was added to 5 mL cell suspension. After vortexing for 2 min, the tube was set aside to rest for 15 min for phase separation. Then, a sample of cell suspension was retrieved from the aqueous phase with a clean Pasteur pipet, while avoiding disturbing the hydrocarbon layer. The concentration of cells initially resuspended in PBS and retrieved from aqueous phase was counted using Z2 particle counter (Beckman Coulter, Miami, FL). Adhesion of cells to the hydrocarbons was evaluated as the fraction partitioned to the hydrocarbon phase (FP = 1− Cf/Co), where Co is the concentration of cells in PBS before mixing and Cf is the cell concentration in aqueous phase after vortexing and phase separation. The larger FP is, the more hydrophobic the membrane surface is.

### Fluorescence recovery after photobleaching (FRAP)

The intracellular trafficking of fluorescently labeled miRNAs after incubating Hela cells and K562 cells with them was investigated in different time points using the fluorescence recovery after photobleaching (FRAP) technique as previously described [24]. Briefly, cells were cultured in a glass bottom dish and were treated with Cy5-miR363 or FAM-miR195 with or without RNAiMAX. FRAP experiments were performed on a Zeiss LSM710 laser confocal microscope (Carl Zeiss, Germany) with 63x/1.4 NA oil-immersion objective. Three annular areas were set as background area, target area and control area. Bleaching was executed in the target area by using 488nm- line for FAM or 633nm- line for Cy5 with 40mW argon laser at 80% power and fluorescence recovery was monitored at lower (4%) laser power. Fluorescence intensity of the bleaching region was measured once per 4 seconds until 460 seconds in K562 cells and once per 6 seconds until 1380 seconds in Hela cells.

### Hydrophilicity analysis of fluorescent dyes

The hydrophilicity of FAM and Cy5 were analyzed using MARVIN BEANS 16.4 (Chemaxon, Hungary) according to their chemical structures. As described in the software manual and reference [25], the Cl^-^ concentration was set to 155 mM and concentration of Na ^+^ and K ^+^ was set to 162 mM and then the LogD value was calculated at pH 7.4. LogD represented the partition coefficient of substance in butanol -n- water and indicated the hydrophilicity, the larger the LogD, the stronger the hydrophobicity.

### Statistical analysis

In this study, all experiments were repeated at least three times. Data were analyzed using SPSS 22.0 (SPSS, Inc., Chicago, IL, USA). The comparison of mean values of different groups was carried out by t test or ANOVA. P value of <0.05 was considered statistically significant.

## Results

### Non-specific binding of fluorescently labeled miRNAs to cell surface independent of sequence

**Fig 1:**
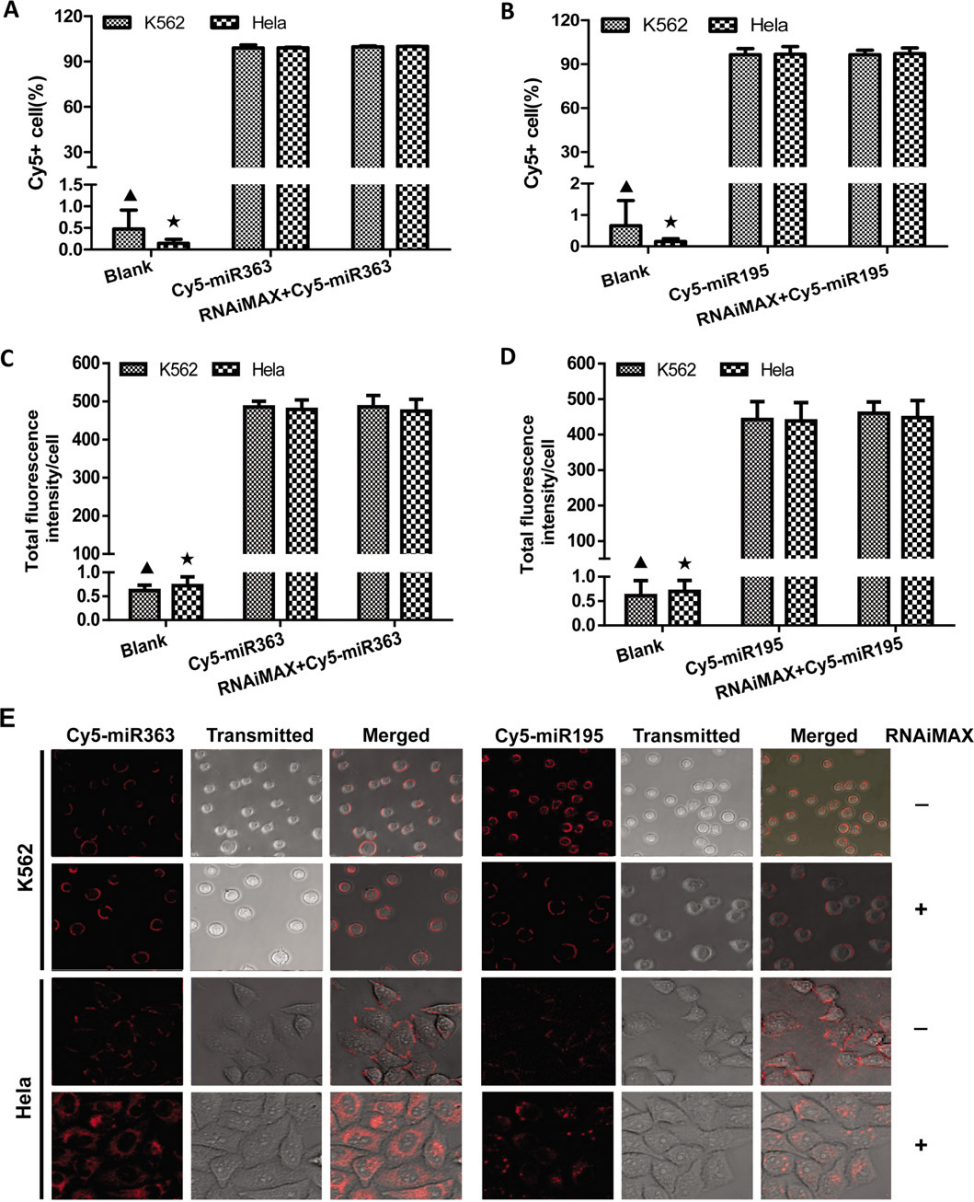
The fluorescently labeled miRNAs could non-specifically bind to cell surface. K562 and Hela cells were separately incubated with Cy5-miR-363/ Cy5-miR-195 with or without RNAiMAX. Then the fluorescence signals of Cy5 on K562 and Hela cells were detected by flow cytometry **(A, B)** and laser scanning confocal microscope (LSCM) without nuclear staining using DAPI **(C, D).** Cy5-positive cells (%) and total fluorescence intensity/cell in each group were calculated and presented in figure. Statistical analysis was performed by ANOVA. ▲: P<0.05 compared with the groups of K562 cells transfected by Cy5-miR363 or Cy5-miR195 with and without RNAiMAX. ★: P<0.05 compared with the groups of Hela cells transfected by Cy5-miR363 or Cy5-miR195 with and without RNAiMAX. (**E**) The K562 and Hela cells with different treatments were scanned by LSCM without nuclear staining using DAPI. The Cy5 fluorescence image, transmitted light image and merged image of them were shown on figure.

MiR-363 or miR-195 labeled with Cy5 was separately transfected into suspension K562 cells using lipofection reagent RNAiMAX. Then the fluorescence signals of Cy5 on K562 cells were analyzed by flow cytometry. The results showed that the percentage of Cy5-positive K562 cells was nearly 100% and this usually implied a nearly 100% transfection efficiency (Fig 1A and 1B). However, this implication was contradictory to the consistent conclusion that suspension cells were difficult to transfect and the transfection efficiency was very low [26, 27]. Therefore, MiR-363 or miR-195 labeled with Cy5 was separately incubated with K562 cells without RNAiMAX. The analysis of flow cytometry data showed similar results (Fig 1A and 1B). Clearly, these results suggested that the abnormally high percentage of Cy5 positive cells was probably not caused by the transfection with RNAiMAX but by the non-specific binding of fluorescently labeled miRNAs to cells. To confirm these results, the fluorescence signals of Cy5 in treated K562 cells were detected by laser scanning confocal microscope without nuclear staining by DAPI. The results were consistent with the above results, namely, the fluorescence signals of Cy5 could be detected in K562 cells treated by Cy5-labeled miR-363 and miR-195 with and without lipofection reagent RNAiMAX. Moreover, there was also no significant difference of TFIPC between different miRNAs with the same treatment (Fig 1C–1E).

### The complex effects of type of fluorescent dyes and cells on the non-specific binding of fluorescently labeled miRNAs to cell surface

Since the transfection efficiency of nucleic acid was significantly different in adherent or suspension cells, Cy5-labeled miR-363 or miR-195 was separately transfected into suspension K562 cells and adherent Hela cells with and without lipofection reagent. Then the fluorescent signals of Cy5 in cells were detected by flow cytometry. The results showed that the percentage of cy5 positive cells in each group of Hela cells treated by Cy5-miR-363 and Cy5-miR-195 was greater than 95% regardless of with or without lipofection reagent. They were similar to the results of suspension K562 cells (Fig 1A and 1B). Moreover, the results of laser confocal microscopy showed that nearly all cells without nuclear staining by DAPI were Cy5-positive and the Cy5 fluorescence signals were in line with the characteristics of membrane staining except for the Hela cells treated with lipofection reagent. The TFIPC was not significantly different between Hela cells and the corresponding K562 cells (Fig 1C–1E).

**Fig 2:**
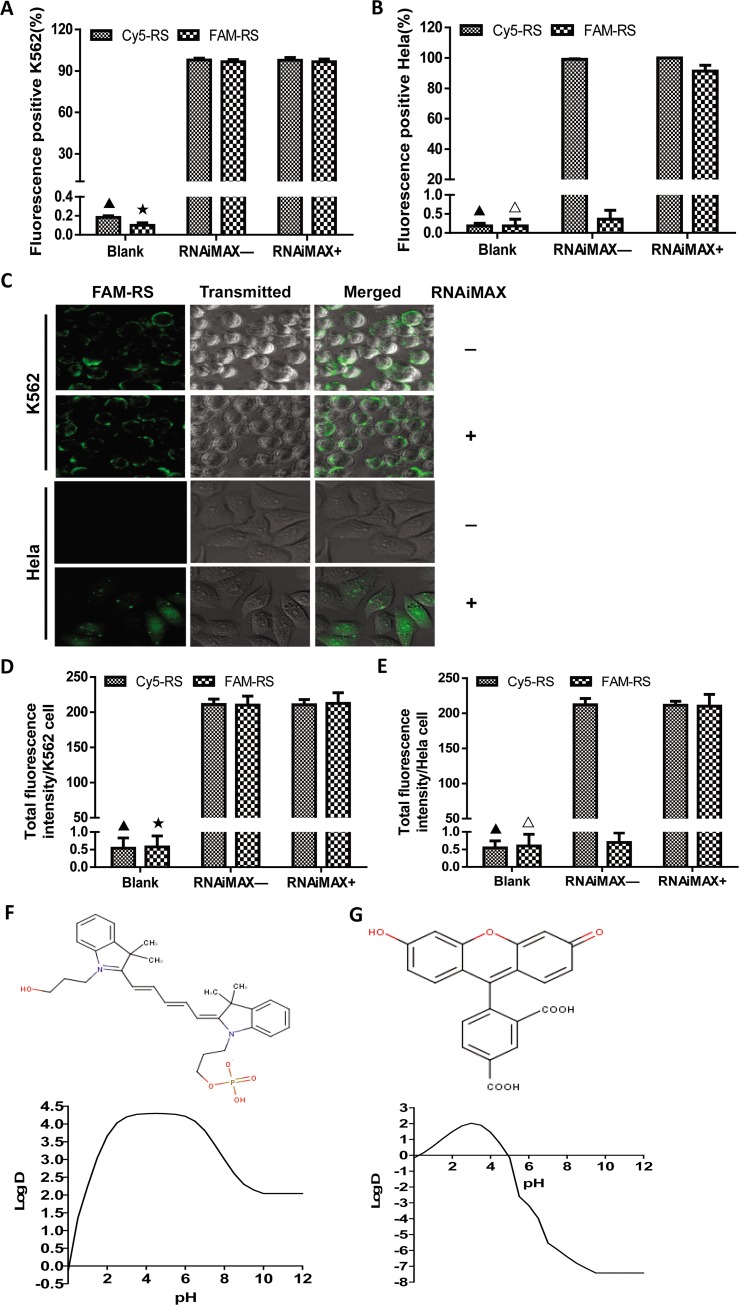
The type of cells and fluorescent dyes influenced on the non-specific binding of fluorescently labeled miRNAs to cell surfaces. The percentage of fluorescence-positive cells in each group of K562 cells **(A)** and Hela cells **(B)** treated by Cy5-RS and FAM-RS with and without RNAiMAX was analyzed using flow cytometry and shown on figure. **(C)** K562 cells or Hela cells were treated by FAM-RS with and without RNAiMAX. The images were scanned by LSCM without nuclear staining using DAPI. The FAM fluorescence image, transmitted light image and merged image of them were shown on figure. The total fluorescence intensity per cell (TFIPC) was also calculated in each group of K562 cells **(D)** and Hela cells **(E)** treated by Cy5-RS and FAM-RS with and without RNAiMAX. All statistical analysis was performed by ANOVA. ▲: P<0.05 compared with the groups of K562 or Hela cells transfected by Cy5-RS with and without RNAiMAX. ★: P<0.05 compared with K562 cells transfected by FAM-RS with and without RNAiMAX. △: P<0.05 compared with Hela cells transfected by FAM-RS and RNAiMAX. The structural formula and hydrophilic curve of Cy5 **(F)** and FAM **(G)** showed that Cy5 was strongly hydrophobic with LogD 3.81 and FAM was strongly hydrophilic with LogD -5.53 at pH = 7.0.

**Fig 3:**
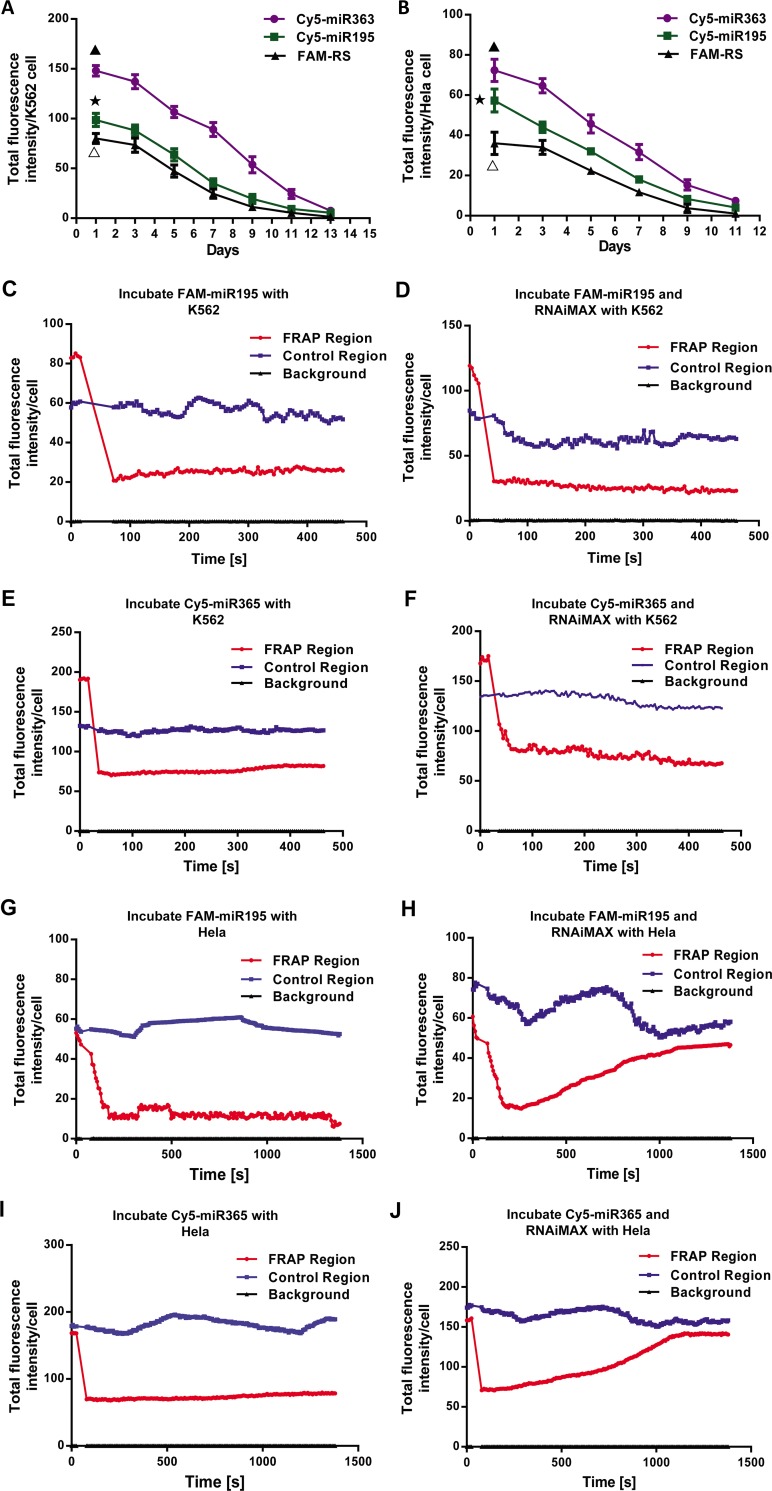
The fluorescence signals of non-specific binding of fluorescently labeled miRNAs were relatively stable and this non-specific binding could not promote miRNAs entering cells. K562 and Hela cells were respectively incubated with Cy5-miR363, Cy5-miR195 and FAM-RS. Then, the total fluorescence intensity per K562 cell (**A**) and Hela cell (**B**) were detected once per two days using laser confocal microscopy. The results were presented as mean ± SD. One-way ANOVA was performed to assess significant differences between the fluorescence intensity of first day and it of other days. P<0.05 compared to the groups of K562 or Hela cells treated with Cy5-miR363 (▲), Cy5-miR195 (★) and FAM-RS (△) in other days. K562 cells were treated with FAM-miR195 (**C**, **D**) or Cy5-miR363 (**E**, **F**) with and without RNAiMAX reagent. Then, FRAP was performed as described in Materials and Methods. Total fluorescence intensity/cell was detected once per 4 seconds until 460 seconds. Results showed that fluorescence signals of the region where the fluorescence had been bleached by high-energy pulsed laser could not recover as time went by in all groups. Hela cells were also treated with FAM-miR195 (**G**, **H**) or Cy5-miR363 (**I**, **J**) with and without RNAiMAX reagent. FRAP was performed and total fluorescence intensity/cell was detected once per 6 seconds until 1380 seconds. In Hela cells treated only with Cy5-miR363 or FAM-miR195, the results were similar to that of K562 cells. In Hela cells treated with Cy5-miR363 or FAM-miR195 and RNAiMAX, fluorescence signals of the region where the fluorescence had been bleached recovered in about 16 minutes because of the high transfection efficiency of RNAiMAX in Hela cells. All results were presented as mean ± SD.

To clarify whether the non-specific binding of fluorescently labeled miRNAs to cells was related to the types of fluorescent dye, the Cy5 and FAM labeled short random sequences, which were the analog of miRNA, were incubated with K562 and Hela cells with and without lipofectamine reagent RNAiMAX. FAM was another commonly used carboxyfluorescein. The results of flow cytometry analysis indicated that the ratios of Cy5-positive or FAM-positive K562 cells were all over 90% and were not significantly different between with and without RNAiMAX. However, the FAM-positive Hela cells were significantly different between with and without RNAiMAX. About 80% Hela cells treated with RNAiMAX and FAM-RS were FAM-positive while Hela cells treated with only FAM-RS were almost FAM negative and had no significant difference compared with control group. The ratios of Cy5-positive Hela cells were all over 90% regardless of RNAiMAX treatment (Fig 2A and 2B). The results of laser confocal microscope were also similar to them of flow cytometry. Namely, TFIPC was not significantly different between K562 cells treated with RNAiMAX+FAM-RS and with only FAM-RS. TFIPC of Hela cells treated with RNAiMAX+FAM-RS was significantly higher than only with FAM-RS (Fig 2C–2E). Additionally, the results of hydrophilic analysis of Cy5 and FAM demonstrated that Cy5 was strongly hydrophobic (LogD = 3.81) and FAM was strongly hydrophilic (LogD = -5.53) at about pH 7.0 (Fig 2F and 2G). Moreover, the signal of non-specific binding of fluorescently labeled miRNAs to K562 or Hela cells was relatively stable. It continued to weaken and was nearly zero until Day 11. (Fig 3A and 3B). Furthermore, the results of FRAP experiments showed that this non-specific binding of fluorescent-labeled miRNAs on cell surface could not promote miRNAs entering cells (Fig 3C–3J). The results of MATH indicated that the membrane surface of K562 was more hydrophobic than it of Hela cells (S1 Fig).

### The important roles of hydrophobic interactions in non-specific binding of fluorescently labeled miRNA to cell surface

**Fig 4:**
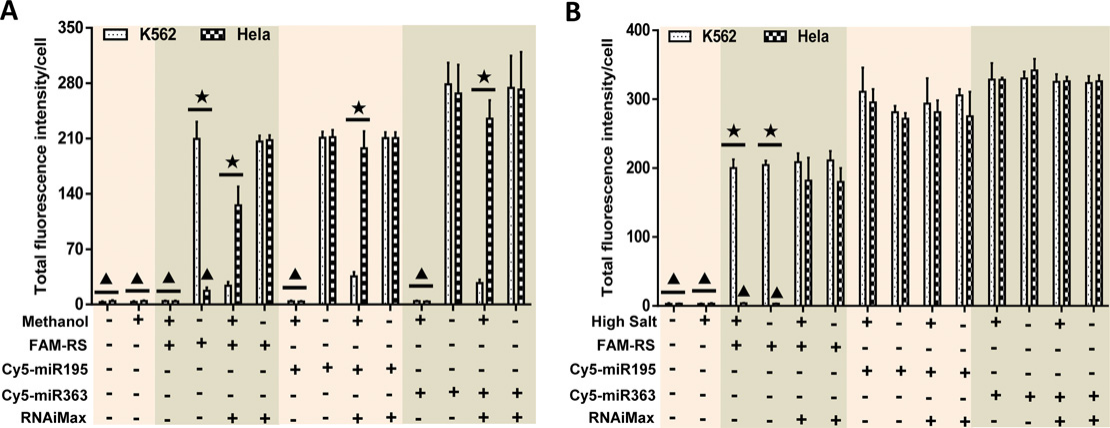
The hydrophobic interaction played important roles in the non-specific binding of fluorescently labeled miRNA to the cell surface. K562 and Hela cells were treated with FAM-RS, Cy5-miR195 and Cy5-miR363 with and without RNAiMAX. Part of them was nuclear-stained by DAPI that dissolved in pure methanol **(A)** or washed by the high salt buffer (cationic and anionic) **(B)** respectively. Then, the fluorescence signals of Cy5 and FAM were detected by LSCM. Total fluorescence intensity/cell of each group was calculated and presented in figure. Statistical analysis was performed by One-way ANOVA. The groups in the same color block were treated with the same fluorescently labeled RNA sequence. ▲: P<0.05 compared with the groups of K562 or Hela cells not indicated by solid black triangle. ★: P<0.05 between the groups of K562 and Hela cells with the same treatment.

Electrostatic and hydrophobic interactions are the most common cause of intermolecular interactions. High salt buffers and organic solvents can destroy them. To reveal the potential mechanisms of non-specific binding of fluorescently labeled miRNA to cell surface, K562 and Hela cells that were treated by Cy5-miR363, Cy5-miR195 and FAM-RS with and without RNAiMAX were nuclear staining using DAPI which was dissolved in pure methanol. The results showed that the fluorescence intensity of cells treated with Cy5 or FAM labeled miRNAs was significantly higher than untreated cells. The TFIPC of K562 cells decreased significantly after nuclear staining using DAPI since the suspension cells were difficult to transfect. However, the fluorescence signals of Cy5 and FAM in Hela cells treated with RNAiMAX and fluorescent-labeled miRNAs was still very obvious after nuclear staining using DAPI. The fluorescence signals of Cy5 and FAM in Hela and K562 cells treated with only fluorescence-labeled miRNAs nearly disappeared after nuclear staining using DAPI (Fig 4A). In addition, the treatment with 4% paraformaldehyde in sample processing had no effect on the results. However, the effect of RNase treatment was similar to that of methanol treatment. Namely, RNase treatment was able to abrogate the non-specific binding in the same way of organic solvent (S2 Fig).

The above groups were treated with high salt buffers that could destroy the electrostatic interactions. The results of laser confocal microscopy showed that a high salt solution had no effect on the non-specific binding of fluorescently labeled miRNAs to cell surfaces. The ratios of positive cells and TFIPC of Cy5 and FAM in K562 cells transfected by Cy5-miR363, Cy5-miR195 and FAM-RS were not significantly different in groups with and without RNAiMAX treatment. The results of Hela cells were similar to the aforesaid results. Namely, TFIPC of FAM in Hela cells without RNAiMAX treatment was almost zero and high salt solution had no effect on this outcome (Fig 4B). Clearly, these results indicated that hydrophobic interactions might play key roles in the non-specific binding of fluorescently labeled miRNAs to the cell surface.

**Fig 5:**
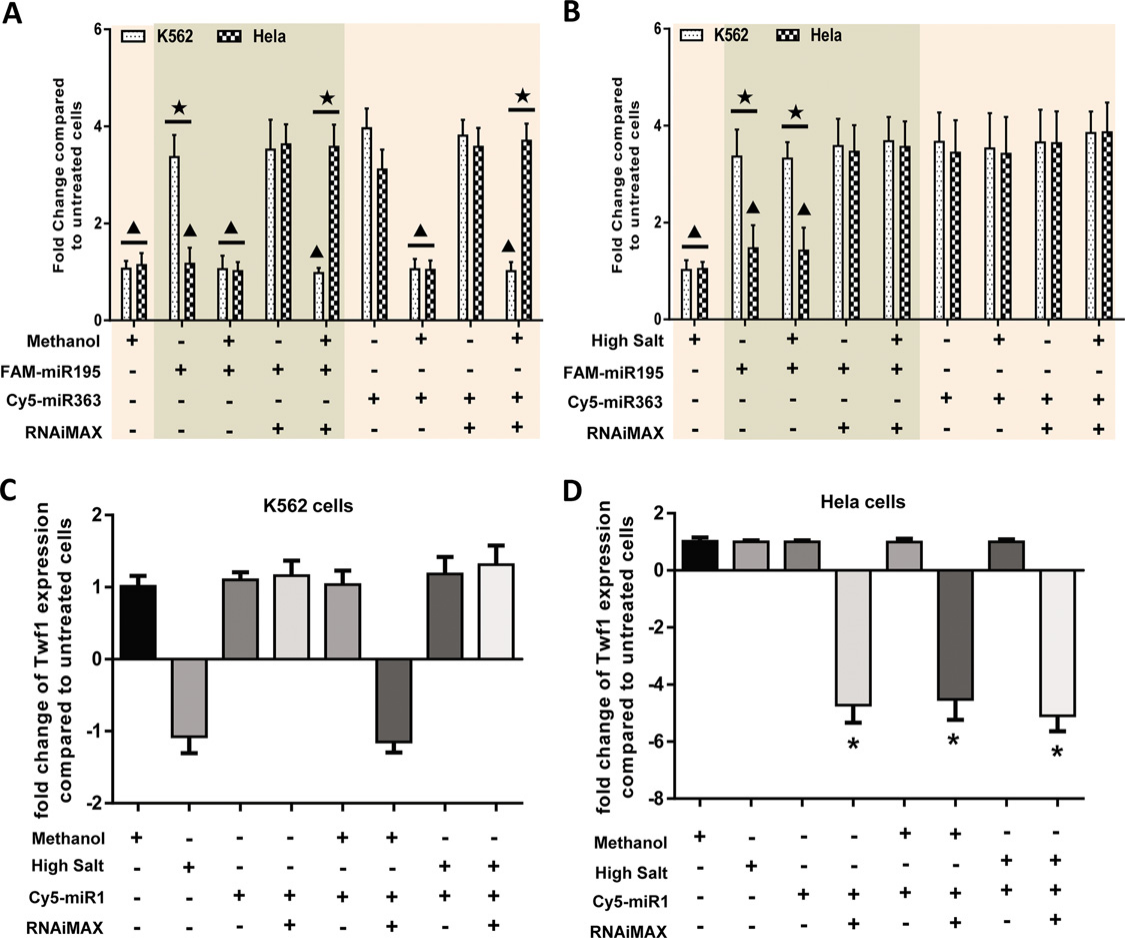
Real time PCR confirmed the important roles of hydrophobic interaction in non-specific binding of fluorescently labeled miRNA to the cell surface. K562 and Hela cells were treated with FAM-miR195 and Cy5-miR363 with and without RNAiMAX. Part of them was washed by methanol or high salt buffer (cationic and anionic) respectively. Then, the amount of these two miRNAs in cells with methanol washing (**A**) and high-salt washing (**B**) was assayed using real time PCR. The groups in the same color block were treated with the same fluorescently labeled RNA sequence. Results were presented as fold change of miRNA expression compared to untreated cells. Additionally, K562 and Hela cells were treated by Cy5-miR1 with and without RNAiMAX. Part of them was also washed by methanol or high salt buffer (cationic and anionic) respectively. Then, Twf1 (target gene of miR1) expression in all groups of K562 cells (**C**) and Hela cells (**D**) was detected using real time PCR. Results were presented as fold change of Twf1 expression compared to the untreated cells. A positive number indicated up-regulation and a negative number indicated down-regulation. All data were shown as mean ± SD. Statistical analysis was performed by One-way ANOVA. ▲: P<0.05 compared with the groups of K562 or Hela cells not indicated by solid black triangle. ★: P<0.05 between the groups of K562 and Hela cells with the same treatment. *: P<0.05 compared with the groups of Hela cells not indicated by asterisk.

To further confirm the above results, K562 and Hela cells that were treated by FAM-miR195 and Cy5-miR363 as described above. Then, the amount of these two miRNAs was assayed using qPCR and luciferase reporter assay. The results of qPCR were consistent with that of Fig 4 (Fig 5A and 5B). The results of luciferase reporter assay showed that the luciferase activity of all groups of K562 cells was not influenced by different treatments and 1-RRR of different treatment groups was not significantly different to each other. This is because the transfection efficiency of RNAiMAX in K562 cells is very low and fluorescent-labeled miRNAs cannot be transfected into K562 cells. On the contrary, the transfection efficiency of RNAiMAX in Hela cells is very high and fluorescent-labeled miRNAs were easily transfected into Hela cells. Therefore, methanol and high salt treatment could not influence the effects of miRNAs that had been transfected into Hela cells. Regardless of treatment of methanol/high salt, 1-RRR of Hela cells treated with fluorescently labeled miRNAs and RNAiMAX reagent was significantly higher than that of other groups of Hela cells. (S3 Fig).

Additionally, the analysis of expression level of miRNA target gene might be another good index of transfection rate to distinguish the non-specific binding. Thus, the expression of Twf1, a target gene of miR1 was analyzed using qPCR. For K562 cells, miR1 could not enter cells and inhibit its target gene expression because of the low transfection efficiency of RNAiMAX. Therefore, although the fluorescence signal in K562 cells treated with Cy5-miR1 was significantly increased compared to untreated cells, Twf1 expression did not change. After treatment with methanol, the fluorescence signal was similar to untreated cells since the non-specifically binding of Cy5-miR1 was washed, whereas the Twf1 expression remained unchanged (S4 Fig and Fig 5C). For Hela cells, the results of cells without treatment of RNAiMAX were similar to that of corresponding K562 cells. However, because of the high transfection efficiency of RNAiMAX in Hela cells, Twf1 expression in Hela cells with treatment of RNAiMAX was significantly decreased regardless of methanol and high salt buffer treatment (S4 Fig and Fig 5D). All these results suggested that the fluorescent-labeled miRNAs could non-specifically bind to cell surface by hydrophobic interaction and organic solvent could reduce the non-specific binding to some extent.

## Discussion

Transfection of exogenous miRNAs is an important technology for functional studies of miRNAs and the transfection efficiency determines the success or failure of the experiments. Many fluorescent dyes can be labeled on nucleic acid sequences and indicate their locations, which can be used to assess the transfection efficiency of cells. Therefore, the non-specific binding of fluorescently labeled nucleic acid sequences to the cell surface would seriously interfere with the results of the assessment. So far, some studies have reported that there were non-specific binding when fluorescently labeled siRNAs or miRNAs were transfected into different cells [17, 18]. The mRNAs labeled with Cy3, which is another cyanine fluorescent dye, could non-specifically bind to the cell surface when they were transfected into Hela cells with lipofectin [19]. Another study found that there were also non-specific binding of fluorescently-labeled proteins to living cells [28]. These results suggested that the non-specific binding of fluorescently labeled biomolecules to the cell surface might be a widespread phenomenon. Our results also indicated the non-specific binding of fluorescent-labeled miRNAs to the cell surface, which was consistent with the previous studies. This study also showed that this non-specific binding was related not to the miRNA sequences but to the types of cells and fluorescent dyes. The non-specific binding of hydrophilic fluorescent dyes (e.g. FAM) was significantly weaker than that of hydrophobic fluorescent dyes (e.g. Cy5) and that in adherent cells which were easily transfected with nucleic acid sequences was also significantly weaker than that in suspension cells which were hard-to-transfect. These studies indicated that it would lead to significant errors in the estimation of transfection efficiency only according to the ratios of fluorescent-positive cells and would interfere with the subsequent experiments because of the non-specific interaction of the fluorescently labeled RNA with cells. However, the aforementioned non-specific interaction was not much reported and fully recognized until now. One of the reasons might be that PCR or Western Blot were often used for assessing transfection efficiency in many studies of miRNA function [29, 30]. Despite this, the ratio of fluorescent-positive cells was still used for assessing transfection efficiency in high-throughput studies in which the transfection efficiency needed to be evaluated quickly [31, 32]. Therefore, the non-specific binding described in this study should be paid more attention and be studied in-depth.

The mechanism studies of non-specific binding of fluorescently labeling miRNAs to cells are still few. Non-specific binding of biological macromolecules is a common phenomenon, which is mainly caused by non-covalent interactions between molecules (such as hydrogen bonding, electrostatic force, hydrophobic force and van der Waals forces, etc). On the other hand, these non-covalent interactions are also important for the specific interactions between biological macromolecules. Of course, the structural adaptation of macromolecules is the basis for ensuring the specificity of interactions. The complexes of positively charged liposomes and negatively charged miRNAs labeled with fluorescent dyes are still positively charged. The cell surface is negatively charged and therefore the positively charged liposome-miRNA complexes can bind to cell surface by electrostatic interactions, then they enter cells through endocytosis. This is the main mechanism of liposomal transfection. However, our study showed that a high salt buffer, which could destroy the electrostatic interactions, could not reduce the non-specific binding of fluorescently labeled miRNA to the cell surface. This indicated that electrostatic interactions might not be the main reason for this non-specific binding. Some studies analyzed the interactions of T47D cells with proteins labeled by Alexa Fluor, Atto and cyanine dyes as well as their chemical properties. They found that hydrophobic fluorescent dyes could non-specifically bind to cells by hydrophobic interactions [28]. Other studies demonstrated that water-soluble fluorescent dyes could also interact with the lipid bilayer [25]. Our study was consistent with the aforementioned results. Namely, the hydrophobic interaction was the important reason for the non-specific binding of fluorescently-labeled miRNA to cells. The treatment of organic solvent could reduce the fluorescence signals caused by the non-specific binding to the cell surface. Moreover, this non-specific binding was closely related to the types of cells and fluorescent dyes. The non-specific bindings of hydrophobic fluorescent dyes to suspension cells were more obvious than that of hydrophilic fluorescent dyes to adherent cells. The mechanism of this difference was very complex. Our results indicated that non-specific binding occurred mainly on cell surface and hydrophobic interactions played a key role in it. Moreover, the surface hydrophobicity of K562 cell membrane was stronger than that of HeLa cell membrane. Therefore, the different hydrophobicity of fluorescent dyes including Cy5 and FAM as well as of the membrane surface of might be involved in the non-specific bindings. Hydrophobic Cy5 could bind more tightly to K562 cells than to Hela cells because of the more hydrophobic surface of K562 cell membrane. Overall FAM is hydrophilic and its hydrophobic groups could still bind to the strong hydrophobic surface of K562 cell membrane, while the binding of FAM to the weak hydrophobic surface of HeLa cell membrane was relatively weak. However, it should be noted that this study only preliminarily investigated the non-specific binding of fluorescently labeled miRNAs in the process of liposomal transfection and the studied fluorescent dyes, nucleic acid sequences and the cell types were relatively few. In fact, more in-depth study on this issue and its mechanism is currently underway in our lab.

In summary, the transfection of miRNA was one of the most commonly used methods for functional studies of miRNAs. The non-specific binding of fluorescently labeled miRNA to cell surface would seriously affect the accuracy and reliability of the results. So far, studies on the mechanism of the above-mentioned non-specific binding have not been reported much and did not attract enough attention. To our knowledge, this study firstly revealed that the fluorescently labeled miRNAs could non-specifically bind to the cell surface by hydrophobic interaction and it was particularly significant in terms of suspension cells and hydrophobic fluorescent dyes. This non-specific binding could be removed by washing cells with organic solvents. Our study implied that the more appropriate fluorescent dyes should be selected in actual experiments according to the studied cell types for ensuring the accuracy of the results.

## Supporting Information

S1 Fig Surface hydrophobicity assay of K562 and Hela cell membrane. K562 and Hela cells were cultured in recommended conditions. Then, cells were harvested and resuspended in PBS. The cell surface hydrophobicity of K562 and Hela cells was evaluated using microbial adhesion to hydrocarbon (MATH) as described in Materials and Methods. Data were presented as mean ± SD. Statistical analysis was performed by t test and p value was shown in figure. FP means the fraction partitioned to the hydrocarbon phase (FP = 1− Cf/Co), where Co is the concentration of cells in PBS before mixing and Cf is the cell concentration in aqueous phase after vortexing and phase separation.
(TIF)

S2 Fig RNase treatment was able to abrogate the non-specific binding in the same way of organic solvent. K562 and Hela cells were incubated with FAM-miR195 and Cy5-miR363 with and without RNAiMAX. Part of them was treated with RNaseA. Then, the amount of these two miRNAs in K562 cells (**A**) and Hela cells (**B**) was assayed using real time PCR. Results were presented as fold change of miRNA expression compared to control, namely, the untreated cells. Additionally, the fluorescence intensity of K562 cells (**C**) and Hela cells (**D**) was also detected by laser confocal microscopy. All data were presented as mean ± SD. All statistical analysis was performed by one-way ANOVA. ▲: P<0.05 compared with the groups of K562 or Hela cells not indicated by solid black triangle.
(TIF)

S3 Fig Luciferase reporter assay confirmed the important roles of hydrophobic interaction in non-specific binding of fluorescently labeled miRNA to the cell surface. The luciferase reporter vector pGL3-miR363 and pGL3-miR195 were separately co-transfected with pRL-TK vector into K562 and Hela cells using Amaxa Nucleofector. Then, the transfected cells were incubated with FAM-miR195 or Cy5-miR363 with or without RNAiMAX reagent. Part of cells was washed by methanol (**A**) or high salt buffer (cationic and anionic) (**B**) respectively. The untreated cells were used as negative control and the cells only co-transfected with pGL3-basic and pRL-TK vector were used as positive control. Luciferase activity was assayed by Dual-Luciferase Reporter Assay System. Results were presented as 1-RRR (Relative Response Ratio). RRR = (firefly/Renilla of experimental sample–firefly/Renilla of negative control)/(firefly/Renilla of positive control–firefly/Renilla of negative control). 1-RRR was positively correlated to the amount of intracellular miRNA. The smaller 1-RRR is, the less intracellular miRNA amount is, and vice versa. Data were presented as mean ± SD. All statistical analysis was performed by one-way ANOVA. ▲: P<0.05 compared with the groups of K562 or Hela cells not indicated by solid black triangle. ★: P<0.05 between the groups of K562 and Hela cells with the same treatment.
(TIF)

S4 Fig Fluorescence signal of K562 and Hela cells treated with Cy5-miR1. K562 and Hela cells were treated by Cy5-miR1 with and without RNAiMAX. Part of them was nuclear-stained by DAPI that dissolved in pure methanol or washed by the high salt buffer (cationic and anionic) respectively. Then, the fluorescence signals of Cy5 were detected by laser confocal microscopy. The total fluorescence intensity per cell of each group was calculated and presented in figure. Data were presented as mean ± SD. Statistical analysis was performed by One-way ANOVA. ▲: P<0.05 compared with the groups of K562 or Hela cells not indicated by solid black triangle. ★: P<0.05 between the groups of K562 and Hela cells with the same treatment.
(TIF)

## References

[1] Guo H, Ingolia NT, Weissman JS, Bartel DP. Mammalian microRNAs predominantly act to decrease target mRNA levels. Nature. 2010; 466: 835–840. 10.1038/nature09267 20703300 PMC2990499

[2] Iorio MV, Ferracin M, Liu CG, Veronese A, Spizzo R, Sabbioni S, et al MicroRNA gene expression deregulation in human breast cancer. Cancer research. 2005;65:7065–7070. 16103053 10.1158/0008-5472.CAN-05-1783

[3] Feng B, Cao Y, Chen S, Ruiz M, Chakrabarti S. miRNA-1 regulates endothelin-1 in diabetes. Life sciences. 2014; 98: 18–23. 10.1016/j.lfs.2013.12.199 24394957

[4] Wang Z, Fan X, Zhang R, Lin Z, Lu T, Bai X, et al Integrative analysis of mRNA and miRNA array data reveals the suppression of retinoic acid pathway in regulatory T cells of Graves' disease. The Journal of clinical endocrinology and metabolism. 2014; 99: E2620–E2627. 10.1210/jc.2014-1883 25233152

[5] Svoboda P. A toolbox for miRNA analysis. FEBS letters. 2015; 589: 1694–1701. 10.1016/j.febslet.2015.04.054 25957774

[6] Martin TM, Wysocki BJ, Beyersdorf JP, Wysocki TA, Pannier AK. Integrating mitosis, toxicity, and transgene expression in a telecommunications packet-switched network model of lipoplex-mediated gene delivery. Biotechnology and Bioengineering. 2014; 111: 1659–71. 25097912 10.1002/bit.25207

[7] Gonzalez G, Pfannes L, Brazas R, Striker R. Selection of an optimal RNA transfection reagent and comparison to electroporation for the delivery of viral RNA. Journal of virological methods. 2007; 145: 14–21. 17561276 10.1016/j.jviromet.2007.04.013 PMC2681243

[8] Kumar VV, Singh RS, Chaudhuri A. Cationic transfection lipids in gene therapy: successes, set-backs, challenges and promises. Curr Med Chem. 2003; 10: 1297–1306. 12678801 10.2174/0929867033457458

[9] Basiouni S FH, Schumann J. High-efficiency transfection of suspension cell lines. BioTechniques. 2012:1–4. 10.2144/000113914 26307260

[10] Christianson HC, Belting M. Heparan sulfate proteoglycan as a cell-surface endocytosis receptor. Matrix biology: journal of the International Society for Matrix Biology. 2014; 35: 51–55. 24145152 10.1016/j.matbio.2013.10.004

[11] Marty C, Meylan C, Schott H, Ballmer-Hofer K, Schwendener RA. Enhanced heparan sulfate proteoglycan-mediated uptake of cell-penetrating peptide-modified liposomes. Cellular and molecular life sciences: CMLS. 2004; 61: 1785–1794. 15241554 10.1007/s00018-004-4166-0 PMC11146021

[12] Abe H, Kool ET. Flow cytometric detection of specific RNAs in native human cells with quenched autoligating FRET probes. Proceedings of the National Academy of Sciences of the United States of America. 2006; 103: 263–268. 16384914 10.1073/pnas.0509938103 PMC1326184

[13] Tu JJ, Rohan S, Kao J, Kitabayashi N, Mathew S, Chen YT. Gene fusions between TMPRSS2 and ETS family genes in prostate cancer: frequency and transcript variant analysis by RT-PCR and FISH on paraffin-embedded tissues. Modern pathology: an official journal of the United States and Canadian Academy of Pathology, Inc. 2007; 20: 921–928. 10.1038/modpathol.3800903 17632455

[14] Moreira BG, You Y, Owczarzy R. Cy3 and Cy5 dyes attached to oligonucleotide terminus stabilize DNA duplexes: predictive thermodynamic model. Biophysical chemistry. 2015; 198: 36–44. 10.1016/j.bpc.2015.01.001 25645886

[15] Sohn SY, Bae WJ, Kim JJ, Yeom KH, Kim VN, Cho Y. Crystal structure of human DGCR8 core. Nat Struct Mol Biol. 2007; 14: 847–853. 17704815 10.1038/nsmb1294

[16] Wang K, Gao Y, Peng X, Yang G, Gao F, Li S, et al Using FAM labeled DNA oligos to do RNA electrophoretic mobility shift assay. Molecular biology reports. 2010; 37: 2871–2875. 10.1007/s11033-009-9841-7 19784797

[17] Grunweller A, Gillen C, Erdmann VA, Kurreck J. Cellular uptake and localization of a Cy3-labeled siRNA specific for the serine/threonine kinase Pim-1. Oligonucleotides. 2003; 13: 345–352. 15000825 10.1089/154545703322617023

[18] Dunne J, Drescher B, Riehle H, Hadwiger P, Young BD, Krauter J, et al The apparent uptake of fluorescently labeled siRNAs by electroporated cells depends on the fluorochrome. Oligonucleotides. 2003; 13: 375–380. 15000828 10.1089/154545703322617050

[19] Barreau C, Dutertre S, Paillard L, Osborne HB. Liposome-mediated RNA transfection should be used with caution. RNA. 2006; 12: 1790–1793. 16921069 10.1261/rna.191706 PMC1581979

[20] Han J, Wang QW, Wang SQ. Fluorescent tag is not a reliable marker for small RNA transfection in the presence of serum. Journal of Biosciences. 2013; 38: 471–478. 23938380 10.1007/s12038-013-9336-5

[21] Mitchell PS, Parkin RK, Kroh EM, Fritz BR, Wyman SK, Pogosova-Agadjanyan EL, et al Circulating microRNAs as stable blood-based markers for cancer detection. Proceedings of the National Academy of Sciences of the United States of America. 2008; 105: 10513–10518. 10.1073/pnas.0804549105 18663219 PMC2492472

[22] Chen X, Ba Y, Ma L, Cai X, Yin Y, Wang K, et al Characterization of microRNAs in serum: a novel class of biomarkers for diagnosis of cancer and other diseases. Cell research. 2008; 18: 997–1006. 10.1038/cr.2008.282 18766170

[23] Zoueki CW, Tufenkji N, Ghoshal S. A modified microbial adhesion to hydrocarbons assay to account for the presence of hydrocarbon droplets. J Colloid Interface Sci. 2010; 344:492–496. 10.1016/j.jcis.2009.12.043 20129613

[24] Sullivan KD, Majewska AK, Brown EB. Single- and two-photon fluorescence recovery after photobleaching. Cold Spring Harb Protoc. 2015 (1): 13–23. 10.1101/pdb.top083519 PMC4399237 25561627

[25] Gruenberg J, Hughes LD, Rawle RJ, Boxer SG. Choose Your Label Wisely: Water-Soluble Fluorophores Often Interact with Lipid Bilayers. PLoS ONE. 2014; 9: e87649 10.1371/journal.pone.0087649 24503716 PMC3913624

[26] Gresch O, Engel FB, Nesic D, Tran TT, England HM, Hickman ES, et al New non-viral method for gene transfer into primary cells. Methods. 2004; 33: 151–163. 15121170 10.1016/j.ymeth.2003.11.009

[27] Yin J, Ma Z, Selliah N, Shivers DK, Cron RQ, Finkel TH. Effective gene suppression using small interfering RNA in hard-to-transfect human T cells. Journal of immunological methods. 2006; 312: 1–11. 16603179 10.1016/j.jim.2006.01.023

[28] Zanetti-Domingues LC, Tynan CJ, Rolfe DJ, Clarke DT, Martin-Fernandez M. Hydrophobic fluorescent probes introduce artifacts into single molecule tracking experiments due to non-specific binding. PLoS One. 2013; 8: e74200 10.1371/journal.pone.0074200 24066121 PMC3774629

[29] Garzon R, Heaphy CE, Havelange V, Fabbri M, Volinia S, Tsao T, et al MicroRNA 29b functions in acute myeloid leukemia. Blood. 2009; 114: 5331–5341. 10.1182/blood-2009-03-211938 19850741 PMC2796138

[30] Zhang GL, Li YX, Zheng SQ, Liu M, Li X, Tang H. Suppression of hepatitis B virus replication by microRNA-199a-3p and microRNA-210. Antiviral research. 2010; 88: 169–175. 10.1016/j.antiviral.2010.08.008 20728471

[31] Liu T, Tang H, Lang Y, Liu M, Li X. MicroRNA-27a functions as an oncogene in gastric adenocarcinoma by targeting prohibitin. Cancer letters. 2009; 273: 233–242. 10.1016/j.canlet.2008.08.003 18789835

[32] Su H, Yang JR, Xu T, Huang J, Xu L, Yuan Y, et al MicroRNA-101, down-regulated in hepatocellular carcinoma, promotes apoptosis and suppresses tumorigenicity. Cancer research. 2009; 69: 1135–1142. 10.1158/0008-5472.CAN-08-2886 19155302

