# Supplementary figures and images for: The Non-Specific Binding of Fluorescent-Labeled MiRNAs on Cell Surface by Hydrophobic Interaction
Source: PLoS One. 2016 Mar 1;11(3):e0149751. doi: 10.1371/journal.pone.0149751 (PMC4773022; doi:10.1371/journal.pone.0149751)

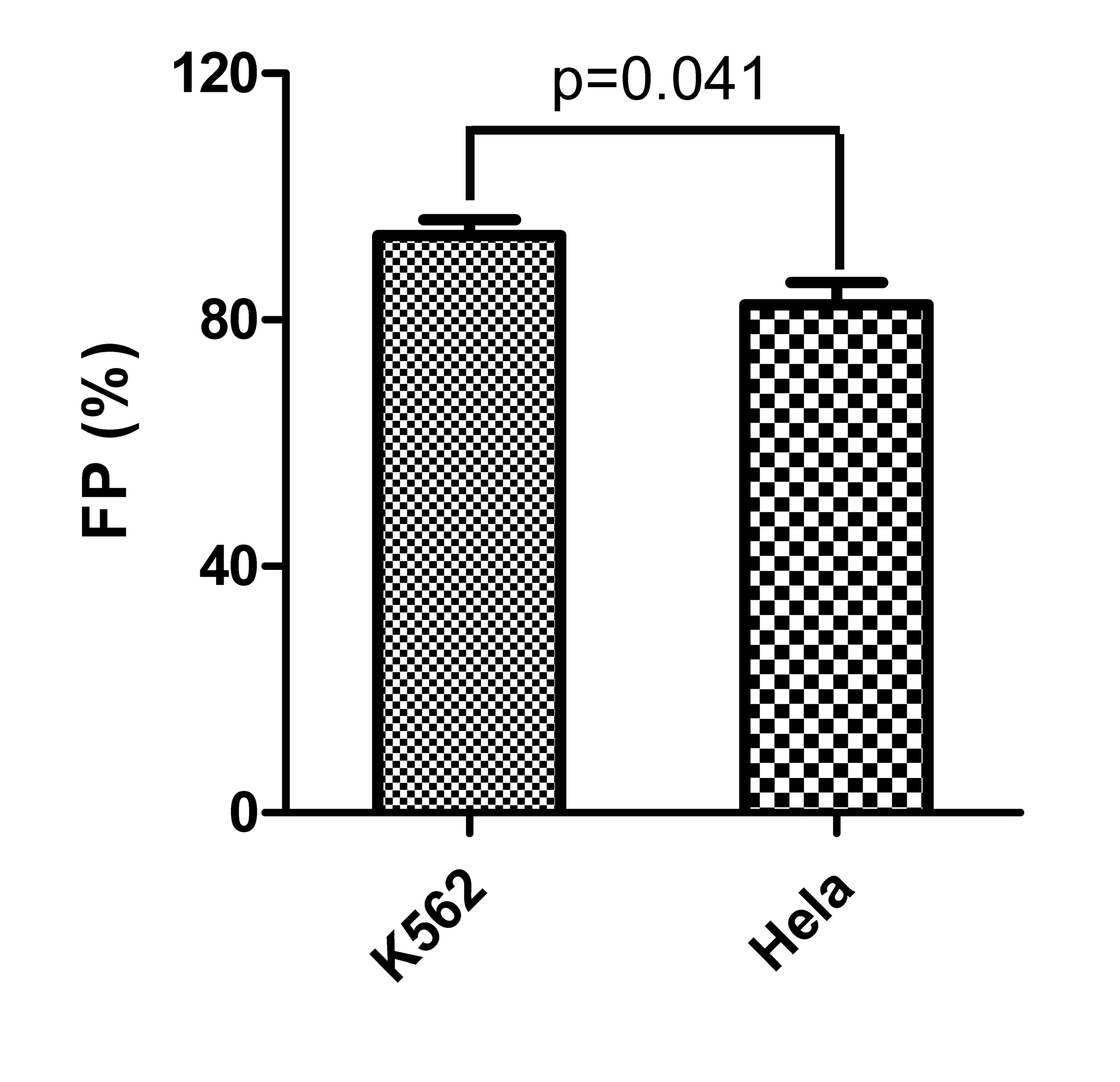

Supplement: S1 Fig — K562 and Hela cells were cultured in recommended conditions. Then, cells were harvested and resuspended in PBS. The cell surface hydrophobicity of K562 and Hela cells was evaluated using microbial adhesion to hydrocarbon (MATH) as described in Materials and Methods. Data were presented as mean ± SD. Statistical analysis was performed by t test and p value was shown in figure. FP means the fraction partitioned to the hydrocarbon phase (FP = 1− Cf/Co), where Co is the concentration of cells in PBS before mixing and Cf is the cell concentration in aqueous phase after vortexing and phase separation. (TIF) [file pone.0149751.s001.tif]

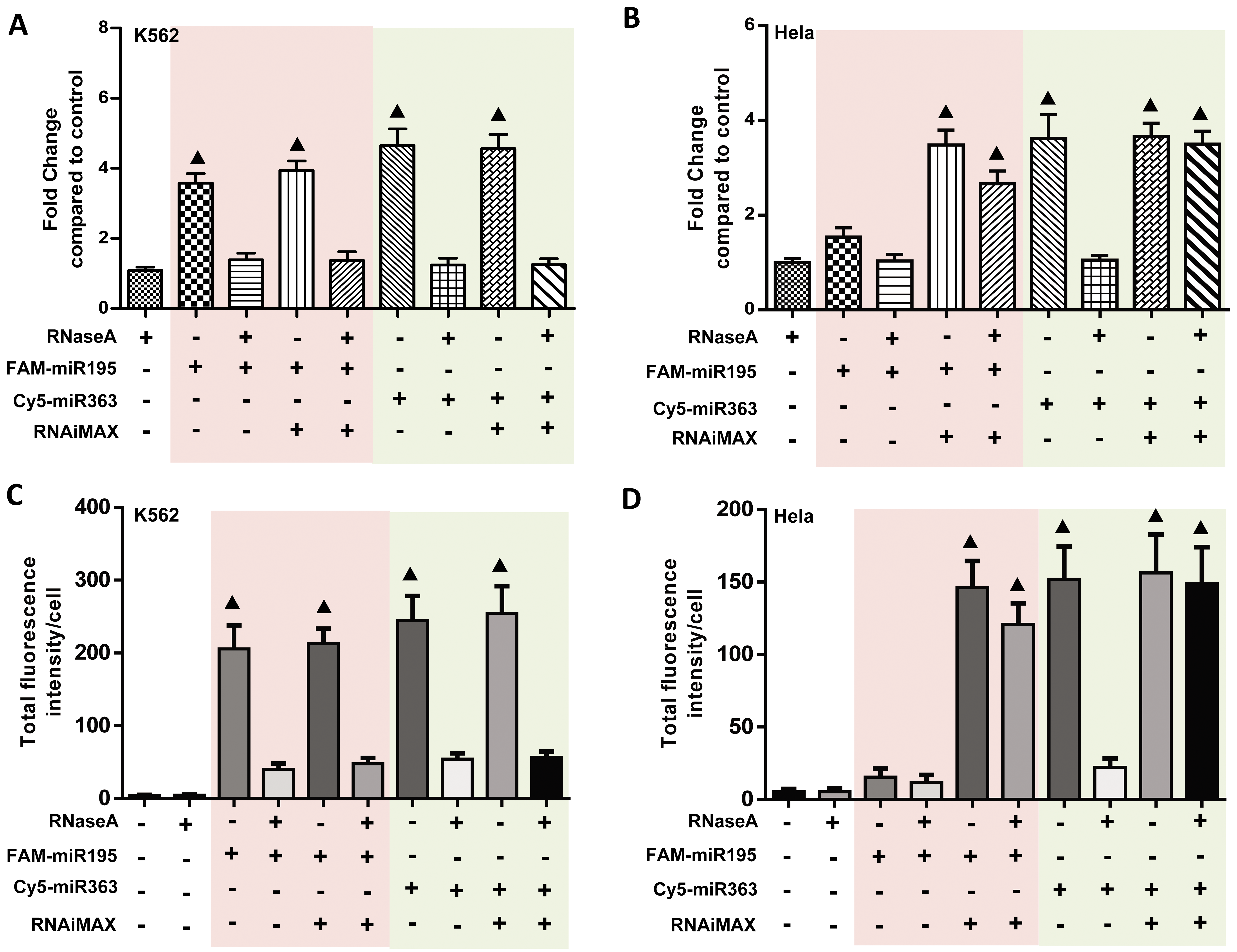

Supplement: S2 Fig — K562 and Hela cells were incubated with FAM-miR195 and Cy5-miR363 with and without RNAiMAX. Part of them was treated with RNaseA. Then, the amount of these two miRNAs in K562 cells (A) and Hela cells (B) was assayed using real time PCR. Results were presented as fold change of miRNA expression compared to control, namely, the untreated cells. Additionally, the fluorescence intensity of K562 cells (C) and Hela cells (D) was also detected by laser confocal microscopy. All data were presented as mean ± SD. All statistical analysis was performed by one-way ANOVA. ▲: P<0.05 compared with the groups of K562 or Hela cells not indicated by solid black triangle. (TIF) [file pone.0149751.s002.tif]

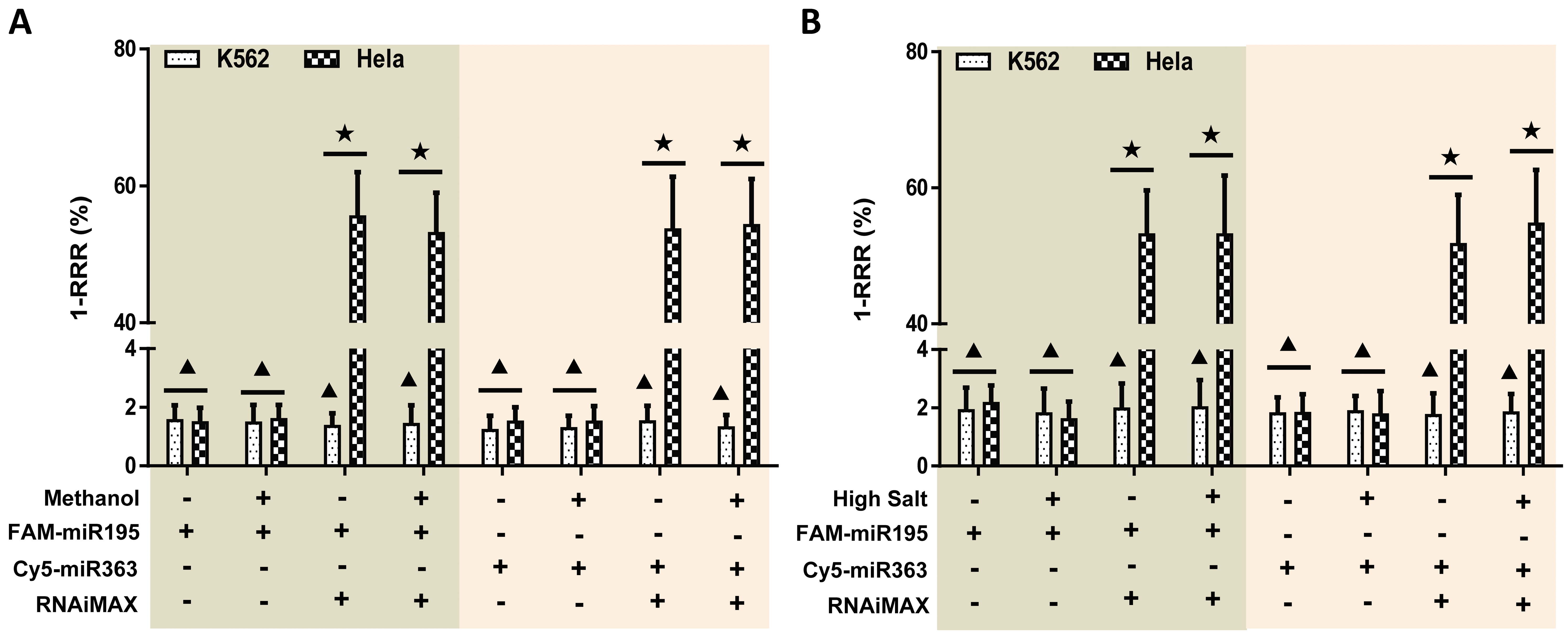

Supplement: S3 Fig — The luciferase reporter vector pGL3-miR363 and pGL3-miR195 were separately co-transfected with pRL-TK vector into K562 and Hela cells using Amaxa Nucleofector. Then, the transfected cells were incubated with FAM-miR195 or Cy5-miR363 with or without RNAiMAX reagent. Part of cells was washed by methanol (A) or high salt buffer (cationic and anionic) (B) respectively. The untreated cells were used as negative control and the cells only co-transfected with pGL3-basic and pRL-TK vector were used as positive control. Luciferase activity was assayed by Dual-Luciferase Reporter Assay System. Results were presented as 1-RRR (Relative Response Ratio). RRR = (firefly/Renilla of experimental sample–firefly/Renilla of negative control)/(firefly/Renilla of positive control–firefly/Renilla of negative control). 1-RRR was positively correlated to the amount of intracellular miRNA. The smaller 1-RRR is, the less intracellular miRNA amount is, and vice versa. Data were presented as mean ± SD. All statistical analysis was performed by one-way ANOVA. ▲: P<0.05 compared with the groups of K562 or Hela cells not indicated by solid black triangle. ★: P<0.05 between the groups of K562 and Hela cells with the same treatment. (TIF) [file pone.0149751.s003.tif]

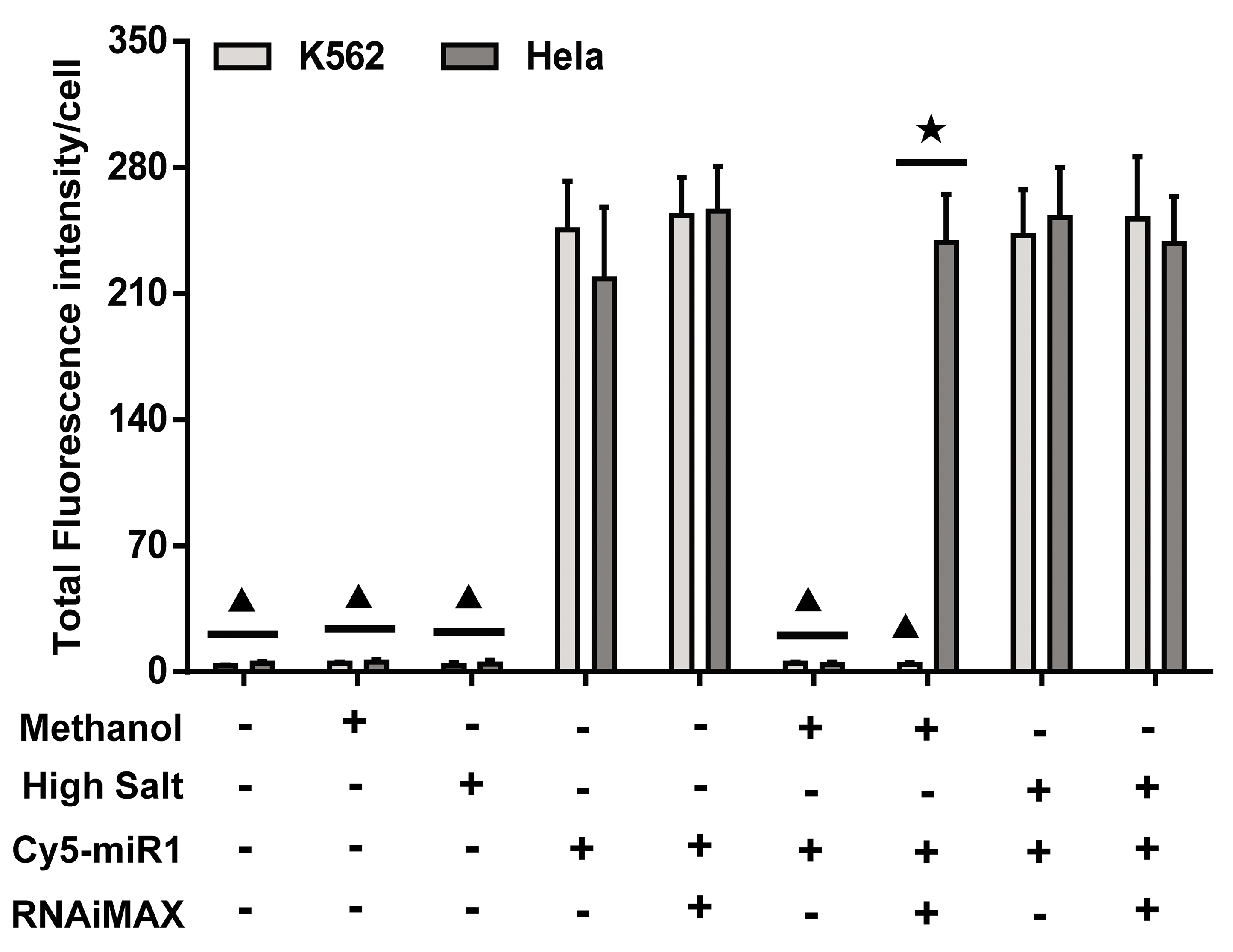

Supplement: S4 Fig — K562 and Hela cells were treated by Cy5-miR1 with and without RNAiMAX. Part of them was nuclear-stained by DAPI that dissolved in pure methanol or washed by the high salt buffer (cationic and anionic) respectively. Then, the fluorescence signals of Cy5 were detected by laser confocal microscopy. The total fluorescence intensity per cell of each group was calculated and presented in figure. Data were presented as mean ± SD. Statistical analysis was performed by One-way ANOVA. ▲: P<0.05 compared with the groups of K562 or Hela cells not indicated by solid black triangle. ★: P<0.05 between the groups of K562 and Hela cells with the same treatment. (TIF) [file pone.0149751.s004.tif]
